# Associations of Sociodemographic and Psychosocial Factors with Headache Symptom Among Indonesian Adolescents Based on the 5th Wave of the Indonesian Family Life Survey (IFLS-5)

**DOI:** 10.34172/jrhs.2023.114

**Published:** 2023-06-29

**Authors:** Jimmy Fransisco Abadinta Barus, Harvey Sudharta, Ika Suswanti

**Affiliations:** ^1^Department of Neurology, School of Medicine and Health Science, Atma Jaya Catholic University of Indonesia, Jakarta, Indonesia; ^2^STIKes Widya Dharma Husada, Tangerang, Indonesia

**Keywords:** Adolescent, Headache, Psychology, Social, Global burden of disease

## Abstract

**Background:** Headaches are common among children and adolescents, with more than half of adolescents reporting headache symptom worldwide. The number of migraine sufferers among adolescents has increased dramatically in the past decade. Headache has negatively influenced children and has been linked with emotional and behavioral problems.

**Study Design:** A cross-sectional study.

**Methods:** This study was conducted using secondary data from the Indonesian Family Life Survey (IFLS) to evaluate the relationship between sociodemographic and psychosocial factors in Indonesian adolescents and headaches. We used data from the fifth wave of IFLS, which was conducted between September 2014 and April 2015. The figures represent roughly 83% of the Indonesian population. We investigated the possible relationship between sociodemographic and psychosocial factors in adolescents with headaches.

**Results:** A total of 3605 participants (1875 females and 1730 males) aged 15 to 19 years with headache symptom were included in the study. Headache was associated with sleep disturbances (OR 1.99; 95% CI: 1.72, 2.30), depression (OR 1.94; 95% CI: 1.65, 2.28), and female gender (OR 1.72; 95% CI: 1.50, 1.98). Other factors contributing to headaches include poor/moderate sleep quality (OR 1.25; 95% CI: 1.08, 1.45) and low income (OR 1.22; 95% CI: 1.01, 1.48).

**Conclusion:** In Indonesian adolescents aged 15 to 19 with headaches, sleep disturbances were the dominant factor associated with headache occurrence. Other factors such as depression, female gender, low socioeconomic status (SES), and poor/moderate sleep quality showed a positive association with headaches but further large population-based studies with more refined variables are needed to elucidate this association.

## Background

 Headaches are prevalent in youngsters, and its prevalence increases during adolescence. Several retrospective investigations found that the prevalence of headache symptoms increased from 37–51% in children aged 7 to 57–82% in teenagers aged 15.^1^ Several school-based cross-sectional studies reported a high prevalence of headaches among children and adolescents globally, with an overall prevalence of 75.7% in Austria, 30.5% in Spain, 36.6% in Germany, 65.9% in Italy, 88% in Croatia, 79.4% in Norway, 49.4% in Japan, and 63% in Taiwan.^2^ There is no national data available on headaches among adolescents in Indonesia. A survey study by Riahta et al in Indonesia showed that only 16.2% of Indonesian adolescents aged 13 to 17 suffer from primary headaches.^3^ According to a study conducted in Korea by Jeong et al on 2466 children and adolescents aged 3 to 18 years, the number of headache sufferers has increased drastically in the last decade, with a three-fold increase in headache prevalence from 2005 to 2016.^4^

 Headaches significantly impact adolescents’ lives, and three-fourths of sufferers continue to experience symptoms throughout adulthood.^5^ Chronic headaches have negatively influenced children, mainly affecting their confidence and life satisfaction.^6^ In adolescents, it has been linked to emotional problems (anxiety and depression), behavioral problems (social impairment and peer interaction), and other somatic symptoms (pain comorbidity and abdominal pain). Several studies have shown the association between primary headaches and lower psychological, physical, emotional, social, and school functioning scores among adolescents compared to headache-free controls.^3,7^

 There is a multidirectional relationship between biological (physiological), psychological (behavioral), and social (environmental) factors in the development of disease and symptoms, including headaches. Each element influences the onset and intensity of headaches in an interrelated manner.^8^ The International Association for the Study of Pain (IASP) defines pain as an unpleasant sensory or emotional experience associated with actual or potential tissue damage.^9^ It is a subjective perceptive phenomenon involving cognitive processing rather than a sensory phenomenon. It rises from multiple brain regions that process different aspects of a pain message. Many of the regions associated with pain processing are involved in psychological processing (like stress, emotions, and attention); therefore, these shared circuits more or less affect each other in some way.^10^ The present study aimed to identify the major sociodemographic and psychosocial factors associated with headache symptoms among adolescents in Indonesia.

## Methods

###  Data source

 The data used in the present study were obtained from the fifth wave of the Indonesian Family Life Survey (IFLS-5). IFLS is a large-scale population-based longitudinal survey designed as a cross-sectional study, collecting a variety of data about socioeconomic factors and health status. It is based on 7224 households with over 22 000 individuals collected from 13 of the nation’s 26 provinces in 1993, representing 83% of the Indonesian population. They used a random stratified sampling method to select 321 enumeration areas in the 13 provinces.^11^ The survey had been conducted five times in 1994, 1998, 2000, and 2008, and the most recent one used in this study was performed between September 2014 and April 2015. The data had been taken from the same individuals from multiple points in time. In IFLS5, the dynasty recontact rate was 92%. The questionnaires used in the IFLS-5 had the same set of questions used in prior waves with some changes and refinements.

###  Sample size

**Figure 1:**
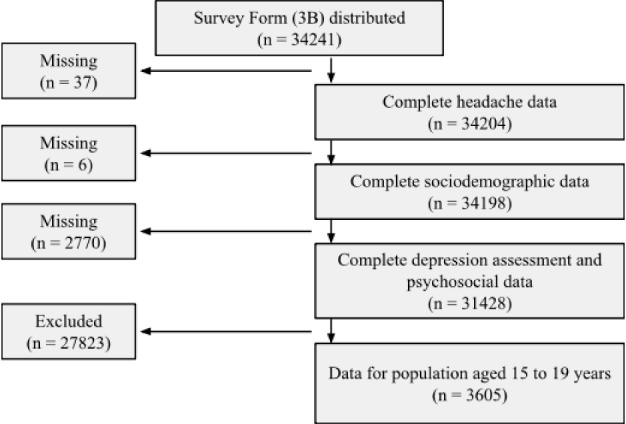
The Sampling Process Used in the Study

 A total of 34241 survey forms (3B) were distributed to the population above 15 years of age. After excluding those who did not meet the age criteria (above 19 years of age) and those with incomplete/missing data, 3605 adolescents aged 15 to 19 years were 3605 adolescents aged 15 to 19 years were left (Figure 1). Of the 3605 adolescents included in the study, 1875 (52%) were female, and 1730 (48%) were male.

###  Study variables

 Headache symptom was the main outcome variable of the present study. The word “symptom” is used because a specific primary headache diagnosis cannot be made. The onset, duration, and characteristics (quality and quantity) of the symptom are needed for clinical diagnosis, which could not be provided by the data. In this survey, participants were asked about acute symptoms (such as headache, fever, flu-like symptoms, abdominal pain, shortness of breath, nausea, and so on) that they had experienced in the last four weeks, using simple “yes” or “no” questions, though some symptoms may have additional information such as a productive or non-productive cough.

 Several variables were considered in this study, including sociodemographic parameters (such as age, gender, education level, and general economic status) and psychosocial components (including smoking and depression). These characteristics were chosen because they were appropriate for the adolescent population, were included in the IFLS survey form, and have long been known to contribute to the development of headaches.

 Age was divided into two categories: 15 to 17 years and 18 to 19. Their age was assessed by asking questions and was confirmed with ID cards or other legal documents. The World Health Organization (WHO) divided adolescence into three phases, including early adolescence (10 to 14 years), middle adolescence (15 to 17 years), and late adolescence (17 to 19 years). Since participants over 15 years of age were included in the survey, only data on middle and late adolescents were available in this study.

 Education level was divided into two categories: ≤ 9 years (primary school or less) and ≥ 9 years (secondary school, college, and university) of education. It was assessed by asking whether the participants had attended school and attained their highest education level. Participants were allowed to respond with primary school, junior high school or the equivalent, senior high school or the equivalent, and college or university (D1, D2, and D3).

 Economic status was classified as “low income” or “middle/high income.” It was evaluated by asking participants to rate their economic situation subjectively. Participants were allowed to respond with numbers ranging from one to six, with one being the lowest and six being the highest. No further action was taken to confirm the economic status. We classified 1–2 as low income, 3–4 as middle income, and 5–6 as high income.

 Depression was divided into two categories: yes and no. The assessment was done using a short version of the Center of Epidemiologic Studies Depression Scale (10-item CES-D Scale).^11^ The full version of the 20-item scale, the CES-D scale, is a self-reported measure of depressive symptoms in the past week optimized for general use in the population. It is designed for studies ascertaining the relationship between depression and other variables. The 10-item CES-D, the short version of CES-D, has an accuracy comparable to that of CES-D, as seen in several studieswith a sensitivity of 91%, specificity of 92%, and a positive predictive value of 92%.^12,13^

 Smoking was divided into two categories: yes and no. The assessment was based on a 22-questions about smoking/chewing tobacco. Several questions were asked, including the age of smoking initiation, method of using, number of cigarettes, urge to smoke, time, and places where they usually smoke.

 Sleep disturbances were divided into two categories: often/sometimes and never. The assessment was based on five retrospective questions over the last seven days. All questions were answered on a five-point ordinal scale indicating agreement or disagreement with the statements. Answers such as always, often, and sometimes were classified as often/sometimes, and answers such as never and rarely were classified as never. Sleep quality followed a similar approach and was divided into two categories: poor/moderate (very bad, bad, moderate) and good (good and very good). It was also assessed using five retrospective questions. All questions were translated and re-translated, referring to PROMIS guidelines.^11^ SurveyMETER (Survey, Measurement, Training, and Research) staff, an independent research organization, did the initial translation into Indonesian.

###  Statistical analysis

 All statistical analyses in the present study were performed using SPSS version 26.0.^14^ There were two main analyses, the bivariate, and multivariate analyses. In the bivariate analysis, we used chi-square as all variables were categorical. In the multivariate analysis, we used logistic regression. A multivariate-adjusted logistic regression model was used to estimate the odds ratios (ORs) and 95% confidence intervals (CI) of the factors associated with headaches. Interaction between variables was evaluated using logistic regression analysis. The correlation between studied variables was calculated and reported using collinearity statistics.

###  Ethics statement

 The data and procedures used in IFLS have been reviewed and approved by the Institutional Review Board (IRBs) in the United States (RAND Corporation) and in Indonesia (Gajah Mada University). All participants had provided written consent before data collection began since the first IFLS. The ethical clearance number obtained from the Human Subjects Protection Committee of the RAND was s0064-06-01-CR01.^13^

## Results

Table 1 shows the characteristics of all adolescent participants that were included in the present study. We found that 56.9% of adolescents experienced headache symptom at least once in the past four weeks. The majority of participants with headache symptom were female late adolescents. Those with headaches were also more likely to be depressed, be more educated, smoke, experience sleep disturbances, and have poor sleep quality.

**Table 1:** Demographic characteristics and psychosocial factors affecting headache among adolescents

**Variables**	**Participants (n=3605)**		**Headache**		***P *** **value**	**Unadjusted OR (95% CI)**
	**Number**	**Percentage**	**Yes**	**No**		
Age (year)						
17-19	2023	56.1	1186	837		1.000
15-16	1582	43.9	864	718	0.017	1.178 (1.031, 1.345)
Gender						
Female	1875	52.0	1189	686		1.000
Male	1730	48.0	861	869	0.001	1.749 (1.531, 1.998)
Education (year)						
≥ 9	2475	68.8	1435	1040		1.000
< 9	1120	31.2	610	510	0.053	1.154 (1.001-1.330)
Economic status						
Low-income	573	16.0	354	219		1.000
Middle-income	3015	84.0	1686	1329	0.011	1.274 (1.061-1.530)
Depression						
Yes	1087	30.2	779	308		1.000
No	2518	69.8	1271	1247	0.001	2.481 (2.129, 2.893)
Smoking						
No	2979	82.6	1704	1275		1.000
Yes	626	17.4	346	280	0.400	1.082 (0.909, 1.286)
Sleep disturbance						
Often/sometimes	1738	48.2	1181	557		1.000
Never	1867	51.8	86	998	0.001	2.435 (2.126, 2.789)
Sleep quality						
Poor/moderate	2282	63.3	1388	894		1.000
Good	1323	36.7	662	661	0.001	1.550 (1.352, 1.777)

Values are presented as numbers (%); the number in parentheses represents the percentage (%) of the total population.

Table 2 shows the association between several sociodemographic and psychosocial factors and headache symptom. The adjusted logistic regression model shows a strong association of headaches with sleep disturbances, depression, and female gender. Other variables including poor/moderate sleep quality and lower income showed a positive association with a slight increase in the OR. Smoking did not show any significant association. The logistic regression analysis showed an interaction between economic status and depression.

**Table 2:** Logistic regression analysis of the factors associated with headache symptom among adolescents aged 15 to19 years

**Variables**	***P *** **value**	**Adjusted OR (95% CI)**
Female	0.001	1.720 (1.498, 1.976)
Lower income	0.039	1.224 (1.011, 1.483)
Depression	0.001	1.937 (1.648, 2.276)
Sleep disturbances (often/sometimes)	0.001	1.988 (1.716, 2.302)
Sleep quality (poor/moderate)	0.002	1.254 (1.083, 1.453)

Table 3 shows the collinearity analysis to observe the correlation between studied variables. The results showed no correlation between the independent variables (VIF score < 10 or tolerance level > 0.01).^15^

**Table 3:** Coefficient estimates of the studied variables associated with headache symptom among adolescents aged 15 to 19 years

	**Unstandardized coefficients**		**Standardized coefficients**		***P *** **value**	**Collinearity statistics**	
**Variables (Ref.)**	**Beta**	**SE**	**Beta**	**t**		**Tolerance**	**VIF**
Constant	0.532	0.071		7.504	0.001		
Age 17-19 years/15-16 years	0.002	0.017	0.002	.091	0.927	0.927	1.079
Female/male	0.142	0.018	0.143	7.810	0.001	0.760	1.316
Education ≥ 9/ < 9	0.032	0.018	0.030	1.798	0.072	0.936	1.069
Low-income/middle-income	0.046	0.022	0.034	2.115	0.034	0.969	1.032
No smoking/smoking	-0.039	0.024	-0.030	-1.616	0.106	0.735	1.360
Depression/no depression	0.143	0.018	0.133	7.910	0.001	0.909	1.100
Sleep disorders (often or sometimes/never)	0.118	0.013	0.158	8.809	0.001	0.799	1.252
Sleep quality (poor or moderate/good)	0.035	0.014	0.044	2.551	0.011	0.849	1.178

^*^Tolerance level > 0.01 and VIF score < 10 indicated that no multicollinearity was observed in the variables.

## Discussion

 In the present study, we investigated several sociodemographic and psychosocial factors associated with headache symptoms among adolescents aged 15 to 19 years in a representative sample of the Indonesian population. A total of 3605 subjects, 1875 females and 1739 males, were included. No study explored the relationship between these factors with headache symptoms among adolescents in Indonesia.

 In the present study, multivariate analysis showed that sleep disturbance had the strongest association with the occurrence of headaches (OR 1.99; 95% CI: 1.72, 2.30), similar to poor sleep quality (OR 1.254; 95% CI: 1.08, 1.45). The American Academy of Sleep Medicine expert panel recommended 8-10 hours of sleep per night. This recommendation was based on general health, cardiovascular health, metabolism, mental health, and longevity consideration. Several large-population surveys, including the Youth Risk Behavior Survey, found that 72.7% of people did not get the necessary amount of sleep. According to the National Sleep Foundation, 62% of undergraduates and 75% of seniors do not get enough sleep.^16^ Some types of headaches are related to sleep (migraine with and without aura, paroxysmal hemicrania, and hypnic headache), and chronic recurring headaches may end up causing sleep disturbance, showing an interdependent relationship. Sleep deprivation or excessive sleep was also thought to be a migraine trigger.^17^ Sleep disturbance may cause instability of the thalamus and other structures (locus coeruleus, ventral part of the periaqueductal gray matter, and dorsal raphe nucleus), thereby reducing pain threshold.^18^ Both sleep disorders and headaches may be symptoms of various common systemic dysfunctions, such as anemia and hypoxemia.^17^

 Depression showed a comparable result with sleep disturbances (OR 1.94; 95% CI: 1.65, 2.28). A large population-based cross-sectional study involving 4872 teenagers aged 12 to 17 years in Norway found that persistent headache was related to anxiety and depression (OR 2.05; 95% CI: 1.61, 2.61). This association was evident across all types of headaches, from migraines to tension-type and non-classifiable headaches. Sleep deprivation has been linked to various negative health and academic outcomes in adolescents. Sleep duration is closely connected with the risk of mood and depression. A study found that sleep deprivation increases bad mood, emotional regulation, and self-harm. The relationship between mood and sleep is complex and bidirectional because poor mood and anxiety can worsen insomnia and vice versa.^19^

 Females were also associated with headaches among adolescents (OR 1.72; 95% CI: 1.50, 1.98). Headaches are more common in girls during the onset of puberty than in boys. Several studies have found that migraines are twice as common in women than in men.^20^ This gender-specific prevalence suggests that hormones play a key role in the pathophysiology of headaches or the mechanism of headache symptoms. The increased number of sufferers after puberty also suggests that hormonal transition after puberty is the key.^21^

 Although two variables, low income and poor/moderate sleep quality, demonstrated a positive association, it lacked clinical relevance, as seen by a modest rise in the OR. Smoking does not appear to be linked to headache symptoms in this population.

 According to American Psychological Association (APA), socioeconomic status (SES) was a key factor influencing the quality of life, especially in children and adolescents. Lower SES in adolescents was associated with physical and psychological health. Lower physical health status was found to be associated with a higher likelihood of being sedentary,^22^ having a higher body mass index, and elevated rates of morbidity and mortality from chronic diseases later in life.^23^ Lower psychological health was associated with higher levels of emotional and behavioral difficulties (social problems, delinquent behavior, and attention deficit), higher rates of mental disorders (anxiety, depression, suicide attempts, drug use/addiction),^22^ and aggression.^23^ All these health issues may be associated with headache symptoms. A study by Stewart et al^24^ found that the prevalence ofmigraine increased as income declined in both males and females, supporting the social causation hypothesis, in which low SES is related to stress and other disease mediators that increase the occurrence and duration of illnesses.

 We compare the actual results of the present study with studies across the globe to observe any similarities or contradictions. Only studies on adolescents in similar age groups were collected. Most studies found a strong association between headaches and insomnia and depression, consistent with the results of the present study.^25,26^ Female gender was associated with unspecified headache in a study^25^, but no association was found in another study^27^; however, their subgroup analysis showed an association between female gender and migraine. This might also be due to the inclusion of children (aged 6–11) in the study, which lowered the overall prevalence as headaches were less prevalent in children. The study by Zewde et al^27^ also showed another contradicting result, reporting that middle to higher income was associated with headaches. A previous study^25^ found tobacco to be associated with headaches, but the current study found no such association. In theory, long-term exposure to nicotine in tobacco may increase pain sensitivity due to neuronal plastic changes and nicotinic acetylcholine receptor desensitization, contributing to the occurrence of headaches.^28^ The non-significant result in the current study could be attributed to the small number of smokers (17.4%) in the study population.

 There are several strengths in the present study. A large adolescent population was included in the study. This study used the latest data from IFLS-5, representing 83% of the Indonesian population in general. This figure is considered high because no national data or surveys on adolescents with headaches are currently available. The information presented in this study may be useful in future population-based studies.^2^ The data used had high reinterview rates, which significantly reduced the risk of bias due to non-random attrition. Standardized protocols and techniques were used in the study. Given that IFLS is an ongoing longitudinal cross-sectional study, the protocols are adjusted over time to improve the data quality, and the assessment tools used have been validated.

 This study has several limitations as well. The headache and other acute symptoms in IFLS data were evaluated by asking if they had these symptoms in the last month. The headache features were not stated. As a result, no definitive diagnosis of primary headache can be made. Psychosocial variables were absent. Several psychosocial challenges in adolescents should be included but not provided in the data set, such as drug abuse, delinquency, and bullying. Additionally, the exclusion criteria were incomplete. Given that the current study relied on secondary data, several neurological diseases that could cause headache were not ruled out. However, the prevalence of neurological diseases among adolescents was thought to be low.

**Highlights**

- More than half (56.9%) of Indonesian adolescents aged 15 to 19 experience headache symptom.
- In Indonesian adolescents aged 15 to 19 with headaches, sleep disturbances were the dominant factor associated with headache occurrence (OR 1.99; 95% CI: 1.72, 2.30).
- Other factors such as depression, female gender, low socioeconomic status, and poor/moderate sleep quality showed a positive association with headaches.

## Conclusion

 In Indonesian adolescents aged 15 to 19 with headaches, sleep disturbances were the dominant factor associated with headache occurrence. Other factors including depression, female gender, low SES, and poor/moderate sleep quality showed a positive association with headaches but further large population-based studies with more refined variables are needed to elucidate this association.

## Acknowledgments

 The data can be accessed publicly thanks to RAND Corporation (http://www.rand.org/labor/FLS/IFLS.html).

## Authors’ Contribution

**Conceptualization:** Jimmy Fransisco Abdinta Barus, Harvey Sudharta, Ika Suswanti.

**Data curation: **Jimmy Fransisco Abadinta Barus, Harvey Sudharta, Ika Suswanti.

**Formal analysis: **Harvey Sudharta, Ika Suswanti.

**Funding acquisition:** Jimmy Fransisco Abdinta Barus, Harvey Sudharta, Ika Suswanti.

**Investigation:** Jimmy Fransisco Abdinta Barus, Harvey Sudharta.

**Methodology:** Jimmy Fransisco Abdinta Barus, Harvey Sudharta, Ika Suswanti.

**Project administration:** Jimmy Fransisco Abdinta Barus, Harvey Sudharta, Ika Suswanti.

**Resources:** Jimmy Fransisco Abdinta Barus.

**Software: **Ika Suswanti.

**Supervision: **Jimmy Fransisco Abadinta Barus.

**Validation:** Jimmy Fransisco Abdinta Barus, Harvey Sudharta, Ika Suswanti.

**Visualization:** Jimmy Fransisco Abdinta Barus, Harvey Sudharta.

**Writing–original draft:** Jimmy Fransisco Abdinta Barus, Harvey Sudharta.

**Writing–review & editing:** Jimmy Fransisco Abdinta Barus, Harvey Sudharta, Ika Suswanti.

## Competing Interests

 The authors have no potential conflict of interests to declare.

## Funding

 This research received no specific grant from any funding agency in the public, commercial, or not-for-profit sectors.
